# Limited Alleviation of Lysosomal Acid Lipase Deficiency by Deletion of Matrix Metalloproteinase 12

**DOI:** 10.3390/ijms252011001

**Published:** 2024-10-13

**Authors:** Martin Buerger, Melina Amor, Alena Akhmetshina, Valentina Bianco, Bianca Perfler, Armin Zebisch, Thomas Weichhart, Dagmar Kratky

**Affiliations:** 1 Molecular Biology and Biochemistry, Gottfried Schatz Research Center, Medical University of Graz, 8010 Graz, Austria; martin.buerger@medunigraz.at (M.B.); melina.amor@medunigraz.at (M.A.); alena.akhmetshina@medunigraz.at (A.A.); valentina.bianco@medunigraz.at (V.B.); 2 Department of Medicine and Surgery, University of Parma, 43126 Parma, Italy; 3 Division of Pharmacology, Otto Loewi Research Center for Vascular Biology, Immunology, and Inflammation, Medical University of Graz, 8010 Graz, Austria; bianca.perfler@medunigraz.at (B.P.); armin.zebisch@medunigraz.at (A.Z.); 4 Division of Hematology, Medical University of Graz, 8010 Graz, Austria; 5 Center for Pathobiochemistry & Genetics, Medical University of Vienna, 1090 Vienna, Austria; thomas.weichhart@meduniwien.ac.at; 6 BioTechMed-Graz, 8010 Graz, Austria

**Keywords:** LAL, lysosomal storage disease, matrix metalloproteinase 12, CD11b+ Ly6G+ cells, myeloid-derived suppressor cells, lymphoid-to-myeloid shift, chronic inflammation

## Abstract

Lysosomal acid lipase (LAL) is the only known enzyme that degrades cholesteryl esters and triglycerides at an acidic pH. In LAL deficiency (LAL-D), dysregulated expression of matrix metalloproteinase 12 (MMP-12) has been described. The overexpression of MMP-12 in myeloid lineage cells causes an immune cell dysfunction resembling that of *Lal* knockout (*Lal* KO) mice. Both models develop progressive lymphocyte dysfunction and expansion of myeloid-derived suppressor (CD11b+ Gr-1+) cells. To study whether MMP-12 might be a detrimental contributor to the pathology of LAL-D, we have generated *Lal/Mmp12* double knockout (DKO) mice. The phenotype of *Lal/Mmp12* DKO mice closely resembled that of *Lal* KO mice, while the weight and morphology of the thymus were improved in *Lal/Mmp12* DKO mice. Cytological examination of blood smears showed a mildly reversed lymphoid-to-myeloid shift in DKO mice. Despite significant decreases in CD11b+ Ly6G+ cells in the peripheral blood, bone marrow, and spleen of *Lal/Mmp12* DKO mice, the hematopoietic bone marrow progenitor compartment and markers for neutrophil chemotaxis were unchanged. Since the overall severity of LAL-D remains unaffected by the deletion of *Mmp12,* we conclude that MMP-12 does not represent a viable target for treating the inflammatory pathology in LAL-D.

## 1. Introduction

Lysosomal acid lipase (LAL) is the sole lipase known to be capable of degrading cholesteryl esters and triglycerides at an acidic pH. It generates both fatty acids and free cholesterol, two essential precursors and signaling molecules. In LAL deficiency (LAL-D), progressive lysosomal accumulation of cholesteryl esters and triglycerides results in diminished abundance of LAL-derived degradation products (i.e., fatty acids). The progressive pathology of LAL-D is characterized by accumulations of lipid-laden macrophages, hepatosplenomegaly, loss of adipose tissue, emphysema, and immune dysfunction. Upon activation, PPARɣ represses the expression of inflammatory cytokines, promoting an anti-inflammatory phenotype of immune cells [1,2]. Conversely, chronic inactivation of PPARɣ signaling in LAL-D leads to the development of a progressive immune cell dysfunction, attributed to the dysregulated production of common myeloid progenitors and granulocyte–macrophage progenitors [3]. This manifests as a progressive lymphoid-to-myeloid shift driven by a systemic expansion of immunosuppressive CD11b+ Gr-1+ cells, which are known as myeloid-derived suppressor cells (MDSCs) [4,5,6] and were shown to inhibit both the proliferation and function of T-cells [3,7]. MDSCs (CD11b+ Gr-1+) can be further categorized into monocytic (CD11b+ Ly6C+) and granulocytic (CD11b+ Ly6G+) MDSCs [4], with the former accounting for the majority of the myeloid expansion in LAL-D [5].

In a myeloid-lineage-specific overexpression of *Mmp12*, similar alterations in the hematopoietic bone marrow progenitor compartment, expansion of CD11b+ Gr-1+ cells, and lymphocyte dysfunction were observed [8]. These results indicated that increased expression of MMP-12 is sufficient to induce a similar immune dysfunction to that observed in *Lal* KO mice, suggesting a harmful contribution of MMP-12 to the pathology and/or progression of LAL-D.

Microarray analysis revealed matrix metalloproteinase 12 (MMP-12) to be the most highly upregulated gene in the lungs of 6-month-old *Lal* knockout (KO) mice [9,10]. MMP-12 was initially characterized as an extracellular matrix (ECM)-degrading enzyme that is secreted by macrophages and is essential for their tissue migration [11]. Only later has MMP-12 been understood to play a broader role in a number of biological functions [12,13,14,15,16]. By degrading ECM proteins, MMP-12 liberates ECM-derived peptides that are collectively referred to as matrikines [17]. Matrikines, like elastin-derived peptides, possess chemoattractant properties [18], promote inflammation [19,20], and induce insulin resistance [21]. Thus, MMP-12 deficiency improved insulin sensitivity, systemic inflammation, and atherosclerosis in a cardiometabolic mouse model [22]. The substrate specificity of MMP-12 is not limited to ECM proteins but also includes various cytokines and cell-surface receptors [23,24], indicating a more complex involvement of MMP-12 in modulating the immune response. The administration of recombinant human MMP-12 via pulmonary injection was sufficient to induce neutrophil infiltration in mice [25]. Likewise, deletion of *Mmp12* resulted in blunted infiltration of alveolar neutrophils and macrophages in smoke-exposed mice [26]. Independent of its proteolytic activities, MMP-12 was also shown to possess transcription factor properties [15,27] that are essential for the release of interferon-γ, thereby facilitating host defense against viral infections.

The similarities between the immune system dysfunctions observed in *Lal* KO and MMP-12-overexpressing mice suggested that MMP-12 may play a role in the pathology of LAL-D. To investigate the extent to which MMP-12 may contribute to LAL-D and whether it represents a viable target for its treatment, we have generated *Lal/Mmp12* double knockout (DKO) mice. We aimed to characterize potential ameliorations, primarily in terms of immune system alterations and secondarily in terms of the broader pathology of LAL-D.

## 2. Results

### 2.1. Deletion of MMP-12 Does Not Alleviate the Phenotype of Lal KO Mice

**Figure 1:**
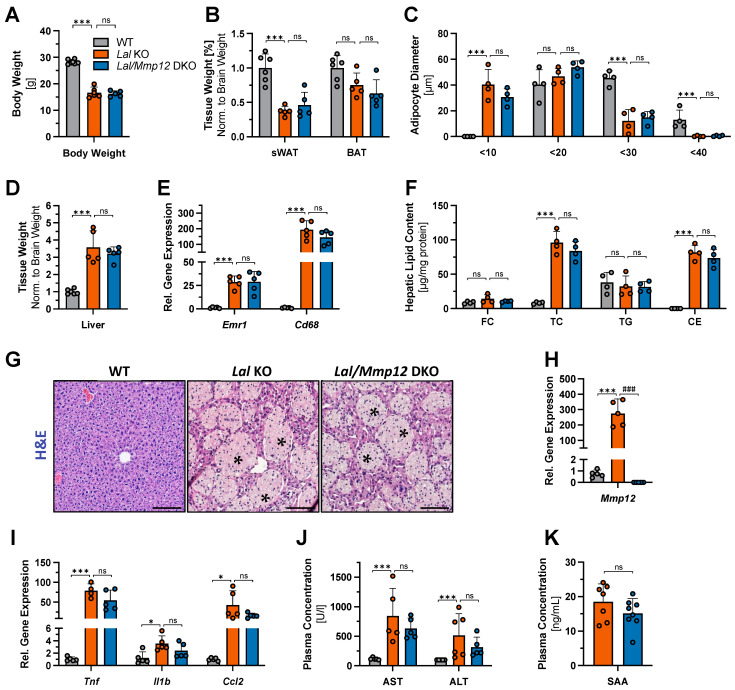
Phenotype of *Lal/Mmp12* DKO mice. Male 30-week-old chow-diet-fed WT, *Lal* KO, and *Lal/Mmp12* DKO mice were fasted for 6 h. (**A**) Body weight. (**B**) Weight of subcutaneous white adipose tissue (sWAT) and brown adipose tissue (BAT), normalized to brain weight; WT mice were arbitrarily set to 1. (**C**) Mean diameter of adipocytes from sWAT paraffin sections. (**D**) Liver weight normalized to brain weight; WT mice were arbitrarily set to 1. (**E**) Hepatic mRNA expression of macrophage markers (*Emr1* and *Cd68*) relative to *Hprt* expression. (**F**) Hepatic lipid parameters. (**G**) Representative images of H&E-stained liver sections. Scale bars, 100 µm. Asterisks indicate the granuloma-like accumulation of lipid-laden macrophages. Hepatic mRNA expression of (**H**) *Mmp12* and (**I**) inflammation/chemotactic markers (*Tnf*, *Il1b*, and *Ccl2*) relative to *Hprt* expression. Plasma concentrations of (**J**) aspartate aminotransferase (AST), alanine aminotransferase (ALT), and (**K**) serum amyloid A (SAA). Data are shown as means (n = 4–8) + SD. ^###^ *p* ≤ 0.001 for the comparison between *Lal* KO and *Lal/Mmp12* DKO mice.

The objective of this study was to evaluate the extent to which MMP-12 contributes to the pathology of *Lal* KO mice. To this end, we generated *Lal/Mmp12* DKO mice and analyzed potential reductions in the severity of LAL-D. At the age of 30 weeks, male *Lal* KO and *Lal/Mmp12* DKO mice exhibited similar reductions in body weight compared to wild-type (WT) mice (Figure 1A). The pronounced adipose tissue loss in *Lal* KO mice, as determined by reduced subcutaneous white adipose tissue (sWAT) and brown adipose tissue (BAT) weights (Figure 1B), as well as decreased mean diameters of white adipocytes (Figure 1C), was also observed in *Lal/Mmp12* DKO mice. This finding indicates that MMP-12 is not involved in adipocyte loss in LAL-D.

The hepatomegaly observed in *Lal* KO mice is primarily driven by the progressive accumulation of cholesteryl ester-filled macrophages [28]. Elevated liver weights (Figure 1D), hepatic expression of the macrophage markers *Emr1* and *Cd68* (Figure 1E), and hepatic cholesteryl ester accumulation (Figure 1F) were comparable between *Lal* KO and *Lal/Mmp12* DKO mice, demonstrating that the extent of hepatomegaly and the number of macrophages were unaffected by the deletion of *Mmp12*. Additionally, we observed no significant differences in the morphology of macrophage aggregates that formed granuloma-like structures (Figure 1G). In view of these results, we conclude that although macrophages are the main source of MMP-12, it is unlikely that MMP-12 impacts macrophages and their dysfunction in LAL-D.

To further explore the relationship between MMP-12 expression and hepatic inflammation in *Lal* KO mice, we assessed hepatocyte health. In accordance with previous reports [9], we found a drastic 275-fold increase in hepatic *Mmp12* expression in *Lal* KO mice (Figure 1H). mRNA expression levels of *Tnf*, *Il1b*, and *Ccl2*, markers for inflammation and macrophage chemotaxis were comparable between the livers of *Lal/Mmp12* DKO and *Lal* KO mice (Figure 1I). Moreover, plasma concentrations of ALT and AST (Figure 1J), indicators of hepatic injury, and of serum amyloid A (Figure 1K), an acute phase marker predominantly produced by the liver, were comparable between both genotypes. These results suggested that MMP-12, similar to its lack of effect on hepatic macrophages, is not involved in mediating overall hepatic pathogenesis in *Lal* KO mice.

### 2.2. Improved Thymus Weight and Morphology in Lal/Mmp12 DKO Mice

**Figure 2:**
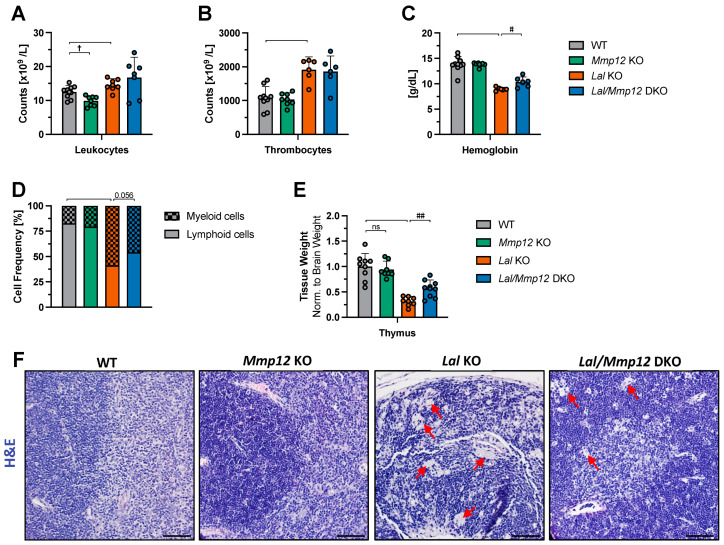
Minor changes in total blood cell counts but ameliorated thymus weight and morphology in *Lal/Mmp12* DKO mice. Complete blood counts in peripheral blood collected from 28–32-week-old chow-diet-fed male WT, *Mmp12*, *Lal* KO, and *Lal/Mmp12* DKO mice for (**A**) total leukocytes and (**B**) thrombocytes. (**C**) Hemoglobin concentrations. (**D**) The frequency of myeloid and lymphoid cells determined by cytological examination of Giemsa-stained blood smears. (**E**) Thymus weight normalized to brain weight; WT mice were arbitrarily set to 1. (**F**) Representative images of H&E-stained thymus sections. Scale bars, 100 µm. Red arrows indicate the accumulation of lipid-laden macrophages. Data are shown as means (n = 4–9) + SD. ^†^ *p* < 0.05 for the comparison between WT and *Mmp12* KO mice; ^#^ *p* < 0.05 and ^##^ *p* ≤ 0.01 for the comparison between *Lal* KO and *Lal/Mmp12* DKO mice.

Both *Lal* KO mice and mice with myeloid-specific overexpression of *Mmp12* showed a strong lymphoid-to-myeloid shift, attributable to both diminished lymphocyte counts and an expansion of CD11b+ Ly6G+ cells [3,8]. To examine whether the deletion of *Mmp12* cures one or both conditions, we characterized immune cells of *Lal/Mmp12* DKO mice. As a control, we also included *Mmp12* KO mice to determine whether potential differences observed in *Lal/Mmp12* DKO were due to MMP-12 deficiency. Previous studies on MMP-12 have focused on its involvement in inflammatory conditions, with no corresponding analysis in non-inflammatory contexts. Complete blood counts of *Mmp12* KO mice revealed a 20.9% decrease in total leukocytes (Figure 2A), originating from a 20.8% decrease in the lymphocyte count and a 21.4% decrease in the granulocyte count (Supplementary Table S1). Furthermore, we observed increased total leukocyte counts in *Lal* KO and *Lal/Mmp12* DKO mice (Figure 2A). The number of thrombocytes was markedly elevated (Figure 2B), whereas hemoglobin levels were significantly reduced in both genotypes compared to WT mice (Figure 2C). A cytological examination of blood smears revealed a slight increase in the frequency of lymphoid cells from 41.1% in *Lal* KO to 54.3% (*p* = 0.056) in *Lal/Mmp12* DKO blood smears (Figure 2D). In addition, the thymus weight was increased 1.8-fold in *Lal/Mmp12* DKO compared to *Lal* KO mice (Figure 2E). Histological examination of hematoxylin and eosin (H&E)-stained thymus sections indicated the absence of the brighter-appearing inner medulla, with only the darker-appearing outer cortex area remaining (Figure 2F). The thymus sections from *Lal* KO mice displayed a pervasive accumulation of lipid-laden macrophages, which were less prominent in *Lal/Mmp12* DKO sections (Figure 2F). These findings indicate an amelioration of the thymus size and morphology by MMP-12 loss in LAL-D.

### 2.3. Decreased CD11b+ Ly6G+ Counts in Bone Marrow, Spleen, and Peripheral Blood of Lal/Mmp12 DKO Mice but Unchanged Composition of the Hematopoietic Progenitor Cell Compartment

**Figure 3:**
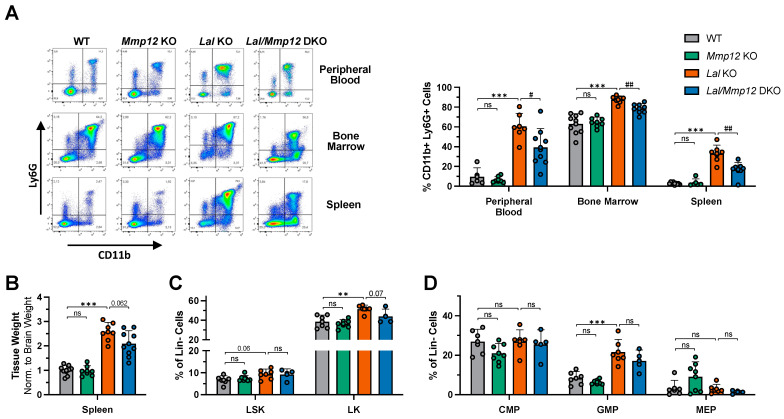
Reduced CD11b+ Ly6G+ counts in blood, bone marrow, and spleen, but no changes in the hematopoietic progenitor compartment of *Lal/Mmp12* DKO mice. (**A**) Representative flow cytometric plots and their quantification, depicting a decreased CD11b+ Ly6G+ fraction in peripheral blood, bone marrow, and spleen of *Lal/Mmp12* DKO compared to *Lal* KO mice. (**B**) Spleen weight normalized to brain weight; WT mice were arbitrarily set to 1. (**C**) The content of Lin- Sca1+ c-Kit+ (LSK) and Lin- c-Kit+ (LK) and (**D**) progenitor cells [common myeloid progenitors (CMPs), granulocyte–macrophage progenitors (GMPs), megakaryocyte–erythrocyte progenitor cells (MEPs)] shown as % of 7-AAD lineage-negative bone marrow cells. Data are shown as means (n = 4–10) + SD. ** *p* < 0.01 and *** *p* ≤ 0.001 for the comparison between WT and Lal KO mice; ^#^ *p* ≤ 0.05 and ^##^ *p* ≤ 0.01 for the comparison between *Lal* KO and *Lal/Mmp12* DKO mice.

Next, we analyzed the composition of cells in the peripheral blood, spleen, and bone marrow by flow cytometry. Compared to *Lal* KO mice, the CD11b+ Ly6G+ fraction in *Lal/Mmp12* DKO mice was markedly decreased from 60.6% to 39.3% in the peripheral blood, from 87.6% to 78.6% in the bone marrow, and from 33.0% to 16.9% in the spleen (Figure 3A). In addition, the spleen weight of *Lal/Mmp12* DKO mice was 19% lower (*p* = 0.062) compared to *Lal* KO mice (Figure 3B). These results suggested that MMP-12 plays a role in the lymphoid-to-myeloid shift in LAL-D.

The expansion of CD11b+ Ly6G+ neutrophils in both *Lal* KO and *Mmp12*-overexpressing mice was attributed to underlying alterations in hematopoiesis [3,8]. We therefore analyzed the composition of hematopoietic bone marrow progenitor cells. No alterations in the Lin- Sca-1+ c-Kit+ fraction was observed in any of the genotypes (Figure 3C). The only significant change was an increase in the Lin- c-Kit+ compartment of *Lal* KO mice (Figure 3C). The common myeloid progenitors as well as the megakaryocyte–erythrocyte progenitor fraction was comparable in all genotypes (Figure 3D). In agreement with previous data [3], the granulocyte–macrophage progenitor fraction was significantly increased in *Lal* KO mice, and comparably elevated levels were found in *Lal/Mmp12* DKO mice (Figure 3D). We therefore conclude that the deletion of *Mmp12* had no effect on the composition of the hematopoietic progenitor cell compartment and is therefore not responsible for the aforementioned lymphoid-to-myeloid reversal.

### 2.4. No Alterations in Neutrophil Chemotaxis and Lymphocyte Markers in the Spleen of Lal/Mmp12 DKO Mice

**Figure 4:**
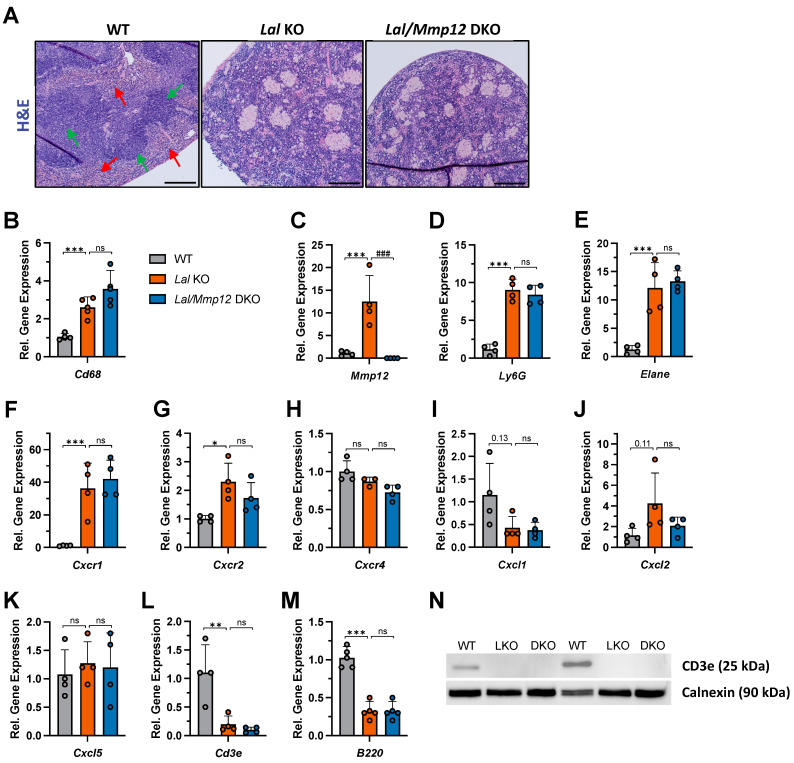
Altered spleen morphology but unchanged neutrophil chemotaxis and lymphocyte markers in *Lal/Mmp12* DKO mice. (**A**) Representative images of H&E-stained spleen sections. Green arrows indicate white pulps; red arrows indicate red pulps. Scale bars, 100 µm. Gene expression of (**B**) the macrophage marker *Cd68*, (**C**) *Mmp12*, (**D**,**E**) neutrophil markers (*Ly6g* and *Elane*), (**F**–**H**) chemokine receptors (*Cxcr1*, *Cxcr2*, and *Cxcr4*), (**I**–**K**) chemokine ligands (*Cxcl1*, *Cxcl2*, and *Cxcl5*), and (**L**,**M**) lymphocyte markers (*Cd3e* and *B220*) relative to *Ppia* expression. (**N**) Immunoblot analysis showing the absence of *CD3e* expression in the spleen of *Lal* KO (LKO) and *Lal/Mmp12* DKO mice. Calnexin was used as loading control. Data are shown as means (n = 3–5) + SD. * *p* ≤ 0.05, ** *p* ≤ 0.01, and *** *p* ≤ 0.001 for the comparison between WT and *Lal* KO mice; ^###^ *p* ≤ 0.001 for the comparison between *Lal* KO and *Lal/Mmp12* DKO mice.

Given that the spleen displayed the most pronounced reduction in CD11b+ Ly6G+ counts among the immune cell compartments examined, we next explored the factors that might explain this tissue-specific observation. Histologically, the spleen is divided into two functionally and anatomically distinct regions known as red pulp and white pulp. As exemplified by the spleen of WT mice, the white pulp is more densely stained by hematoxylin, while the red pulp appears brighter and less dense (Figure 4A). This morphological distinction was absent in both *Lal* KO and *Lal/Mmp12* DKO mice. Similar to the liver (Figure 1G), there was also a comparable accumulation of lipid-laden macrophages in the spleen of *Lal* KO and *Lal/Mmp12* DKO (Figure 4A), consistent with an equivalent expression of the macrophage marker *Cd68* (Figure 4B). The expression of *Mmp12* was only increased 13-fold in the spleen of *Lal* KO mice (Figure 4C), compared to the 275-fold increase observed in the liver (Figure 1H). The expression of the two neutrophil-specific markers *Ly6G* and *Elane* was equally increased in *Lal* KO and *Lal/Mmp12* DKO spleens (Figure 4D,E), as were the mRNA expression levels of *Cxcr1* and *Cxcr2*, two major receptors for neutrophil activation and chemotaxis (Figure 4F,G). Similarly, the expression of *Cxcr4*, a chemokine receptor known to regulate tissue exfiltration and bone marrow homing in aged neutrophils [29], showed no changes across all three genotypes (Figure 4H). In mice, CXCR1 and 2 have three ligands (*Cxcl1*, *Cxcl2,* and *Cxcl5)*, which were all unaffected by LAL or LAL/MMP-12 deficiency (Figure 4I–K). Thus, these data suggested that the chemotaxis of neutrophils was unaltered by the deletion of *Mmp12* and therefore cannot explain the reduced number of CD11b+ Ly6G+ cells in the spleen of *Lal/Mmp12* DKO mice.

One potential explanation for the absence of phenotypic amelioration by *Mmp12* deletion could be the compensatory upregulation of other MMP family member(s). The reduced expression of *Mmp3* and *Mmp13* without upregulation of any MMP argued against compensation of these proteins in the spleen of *Lal/Mmp12* DKO mice (Supplementary Figure S1). Whether another as yet unknown protein compensates for the deletion of *Mmp12* remains to be determined.

The spleen is an important secondary lymphatic organ facilitating the interaction of both T-cells and B-cells. The mRNA expression of *Cd3e* and *B220*, specific markers for T-cells and B-cells, respectively, was strongly diminished in *Lal* KO spleens and did not recover in *Lal/Mmp12* DKO mice (Figure 4L,M). In accordance, the protein expression of CD3e was undetectable in the spleen of both genotypes (Figure 4N), further indicating that the reduction in CD11b+ Ly6G+ cells did not lead to detectable improvements in the spleen pathology of *Lal/Mmp12* DKO mice.

## 3. Discussion

LAL-D has been shown to cause drastically increased expression of MMP-12 in the lungs and in CD11b+ Gr-1+ cells [8,9]. Mice with overexpression of MMP-12 specifically in myeloid lineage cells established a causal relationship between MMP-12 expression and the development of both a lymphocyte dysfunction and an expansion of CD11b+ Ly6G+ cells [8], thereby mimicking parts of the clinical setting of LAL-D [3]. The authors concluded that MMP-12 overexpression causes an abnormal development of hematopoietic progenitor cells, favoring the myeloid lineage [8]. To study the contribution of MMP-12 to immune cell dysfunction in LAL-D, we generated *Lal/Mmp12* DKO mice. As expected, hallmarks of LAL-D, including lysosomal lipid accumulation in the liver or adipose tissue loss, were not improved in the DKO mice. However, pathologies observed in *Mmp12*-overexpressing mice, such as immune dysfunctions, may have been alleviated in *Lal/Mmp12* DKO mice. Although MMP-12 inactivates an array of chemokines (CXCL2, CCL2, -7, -8, and -13), thereby worsening lipopolysaccharide-stimulated infiltration of neutrophils and macrophages in *Mmp12* KO mice [23], neither marker genes for hepatic macrophage accumulation nor macrophage recruitment were affected. The contribution of MMP-12 to hepatic monocyte infiltration and macrophage accumulation in *Lal* KO mice is likely irrelevant compared to the pathologic lipid accumulation in macrophages and hepatocytes.

Several indicators for reduced immune system pathology were observed in *Lal/Mmp12* DKO mice. Most notably, the size and morphology of the thymus, a primary lymphoid organ essential for the maturation of T-cells, were markedly improved. The unchanged number of circulating lymphocytes and expression of the T- and B-cell markers *Cd3e* and *B220* in the spleen, however, suggested that an ameliorated thymus pathology did not affect lymphocyte numbers in *Lal/Mmp12* DKO mice. Persistent, unresolved inflammation in *Lal* KO mice causes the development of CD11b+ Ly6G+ MDSCs [5,30]. The immune-suppressive properties of MDSCs were shown to inhibit the proliferation and function of T-cells [4,6,7,8], thereby contributing to the development of severe pathologies across multiple organs. Accordingly, a reduction in CD11b+ Ly6G+ MDSCs may provide a plausible explanation for the observed improvement in thymus weight and morphology of *Lal/Mmp12* DKO mice. In addition to lymphocyte dysfunction, the expansion of myeloid cells contributes to the lymphoid-to-myeloid shift observed in *Lal* KO mice [3,6]. A combination of cytological examination of blood smears and immunophenotyping by flow cytometry indicated a partial reversal of the myeloid shift in the immune cell compartment of the peripheral blood, bone marrow, and spleen in *Lal/Mmp12* DKO mice. The finding that the deletion of *Mmp12* resulted in a reduction in myeloid expansion, while *Mmp12* overexpression caused its increase, provides evidence that MMP-12 directly contributes to the myeloid expansion in LAL-D. As LAL-D is a highly complex chronic inflammatory condition, MMP-12 can at best be considered one pro-inflammatory factor among many.

Remarkably, the immune system alterations observed in *Lal* KO and *Mmp12*-overexpressing mice share underlying changes in hematopoietic progenitor cells. However, comparable progenitor cell populations in *Lal* KO and *Lal/Mmp12* DKO mice indicated that MMP-12 itself does not affect the bone marrow progenitor cell compartment in LAL-D. In agreement, *Mmp12* mRNA is only very slightly expressed (Ct value > 33) in the bone marrow of WT and *Lal* KO mice (data not shown). The absence of a myeloid cell expansion in lung-epithelial-cell-specific *Mmp12*-overexpressing mice [10] further suggests that peripherally secreted MMP-12 has no direct effect on bone marrow hematopoiesis. In contrast, myeloid-cell-specific *Mmp12* overexpression affected hematopoiesis because MMP-12 was produced by myeloid cells in the bone marrow.

MMP-12 has been shown to modulate neutrophil chemotaxis [20,23,25,31]. An injection of recombinant human MMP-12 resulted in an early neutrophil recruitment [25], while the application of an MMP-12 inhibitor blunted alveolar neutrophil recruitment following smoke exposure [20]. Similarly, in a mouse model of myocardial infarction, prolonged inflammation in *Mmp12* KO mice resulted in a worsened neutrophil influx [31]. Despite these findings, we failed to observe any significant differences in the expression of chemokines or chemokine receptors in *Lal/Mmp12* DKO mice. Gene expression of the neutrophil markers *Ly6g* and *Elane* remained unchanged in the *Lal/Mmp12* DKO spleen, whereas flow cytometry yielded reductions in CD11b+ Ly6G+ neutrophil counts across several immune cell compartments. These two observations are not mutually exclusive, because qPCR quantifies the total abundance of a given mRNA, whereas flow cytometry provides a semi-quantification of the percentage of cells expressing a particular surface protein. We speculate that the deletion of *Mmp12* might have altered neutrophil maturation, activation, or viability [32], thereby leading to disproportional changes between neutrophil numbers and *Ly6g* expression. Whether the absence of a more pronounced improvement of the phenotype in *Lal/Mmp12* DKO mice might be explained by a compensatory upregulation of a so far unknown protein is elusive. Other MMP family members, however, were not upregulated in the spleen of *Lal/Mmp12* DKO mice.

Taken together, our results demonstrated a limited contribution of MMP-12 to the immune dysfunction in LAL-D. *Lal/Mmp12* DKO mice displayed improvements in thymus weight and morphology and a mild reversal of the lymphoid-to-myeloid shift across different immune cell compartments. Notably, the overall severity of LAL-D remains unaffected by the deletion of *Mmp12.* Therefore, we conclude that MMP-12 is not a viable therapeutic target for the treatment of LAL-D in mice.

## 4. Materials and Methods

### 4.1. Animals

*Lal* KO mice [33] were backcrossed onto the C57BL/6J background and crossed with *Mmp12* KO mice (C57BL/6J background) (JAX#004855; The Jackson Laboratory, Bar Harbor, ME, USA) to generate *Lal/Mmp12* DKO mice. All mice were maintained in a clean environment with a regular light–dark cycle (12 h/12 h) on a standard chow diet. Animal experiments were carried out in accordance with the EU Directive 2010/63/EU and approved by the Federal Ministry of Science, Research, and Economy, Vienna, Austria (BMBWF-66.010/0138-V/3b/2019).

### 4.2. Sample Collection

At around 30 weeks of age, male mice were sacrificed after a 6 h fasting period. Peripheral blood was collected by cheek puncture into EDTA collection tubes. Whole blood was used for a complete blood cell count using a V-sight (Menarini diagnostics, Florence, Italy) and for cytological examination of Giemsa-stained blood smears (Merck, Darmstadt, Germany). Plasma was isolated by centrifugation of blood at 6300*× g* (10 min, 4 °C) and stored at −20 °C. The organs were collected after cervical dislocation, weighed, immediately frozen in liquid N_2_, and stored at −80 °C. The femur, tibia, and spine were harvested and kept in ice-cold 1× PBS until further processing.

### 4.3. Hematoxylin and Eosin (H&E) Staining

Freshly isolated adipose tissue, spleen, thymus, and liver tissues were fixed in 10% formalin for 12 h and stored in PBS until paraffin embedding. The paraffin blocks were sectioned into 5 µm thick sections, deparaffinized, and stained with H&E. The mean adipocyte diameter of sWAT sections was analyzed using Fiji Software [34] and the Adiposoft plugin [35].

### 4.4. Protein Isolation and Immunoblotting

Fifty milligrams of frozen spleen tissue was lysed in 100 µL of RIPA buffer containing protease/phosphatase inhibitor cocktail (PIC) (1:1000; Merck, Darmstadt, Germany). After centrifugation at 16,000× *g* and 4 °C for 30 min, protein concentrations of the supernatants were estimated (DC Protein assay, Bio-Rad Laboratories, Hercules, CA, USA). Then, 100 µg of protein was separated by SDS-PAGE and transferred onto a PVDF membrane. The membrane was incubated with a rabbit polyclonal anti-calnexin antibody (1:1000, #2679T; Cell Signaling Technology, Danvers, MA, USA) and a rabbit monoclonal anti-CD3e antibody (1:1000, #78588; Cell Signaling Technology). HRP-conjugated goat anti-rabbit antibody (1:2500, #31460; Thermo Fisher Scientific, Waltham, MA, USA) served as a secondary antibody before detection by enhanced chemiluminescence on a ChemiDoc MP imaging system (Bio-Rad Laboratories). CD3e was quantified by densitometry (Fiji Software [34]) and normalized to the expression of calnexin as loading control.

### 4.5. RNA Isolation, Reverse Transcription, and Real-Time PCR

RNA from liver and spleen tissue was isolated using the TriFast reagent (Peqlab, Erlangen, Germany) according to the manufacturer’s protocol. A mass of 2 µg of RNA was reverse transcribed using the High Capacity cDNA Reverse Transcription Kit (Applied Biosystems, Carlsbad, CA, USA). qRT-PCR was performed on a Bio-Rad CF X96 real-time PCR system (Bio-Rad Laboratories, Hercules, CA, USA) using GoTaq^®^ qPCR Mastermix (Promega, Madison, WI, USA). Samples were analyzed in duplicate and normalized to the expression of cyclophilin A *(Ppia)* or hypoxanthine phosphoribosyltransferase ***(****Hprt)*. The primer sequences are listed in Supplementary Table S2.

### 4.6. ALT, AST, and SAA Measurements

Plasma alanine aminotransferase (ALT) and aspartate aminotransferase (AST) levels were quantified using GOT/AST-PIII Fuji Dri-Chem slides (#3150) and GPT/ALT-PIII Fuji Dri-Chem slides (#3250) and analyzed with a Fuji Dri-chem NX500 (Fujifilm, Tokyo, Japan). Serum amyloid A concentrations were quantified by ELISA according to the manufacturer’s instructions (DY2948-05; Bio-Techne, Minneapolis, MN, USA).

### 4.7. Tissue Lipid Extraction and Quantification

Pieces of liver tissue with a mass of 50 mg in 100 µL of lysis buffer (100 mM potassium phosphate, 250 mM sucrose, 1 mM EDTA, pH 7) were sonicated for 2 × 15 s on ice. After centrifugation at 16,000× *g* and 4 °C for 30 min, the protein concentrations were estimated at 650 nm (DC Protein assay, Bio-Rad Laboratories, Hercules, CA, USA). Lipids were extracted from a volume corresponding to 1 mg of protein using the Folch method. Free cholesterol (#1132300F, Greiner, Kremsmünster, Austria), total cholesterol (#113009910023; DiaSys Diagnostic Systems, Holzheim, Germany), triglyceride (#157600010023, DiaSys Diagnostic Systems), and free fatty acid (Wako Chemicals, Richmond, VA, USA) concentrations were determined by spectrophotometric assays at 490 nm. Cholesteryl ester concentrations were calculated by subtracting free cholesterol from total cholesterol values.

### 4.8. Flow Cytometry

To isolate the bone marrow, the femur, tibia, and spinal cord were crushed using a mortar and pestle and rinsed through a 70 µm cell strainer with 1× PBS containing 10% FCS. Next, 50 µL of peripheral blood was aliquoted, ~30 mg of the spleen was squeezed through a 70 µm cell strainer, and bones were crushed with mortar and pestle. All samples were treated with an ammonium chloride–potassium buffer to lyse red blood cells and washed with HEPES-buffered saline containing 10% fetal calf serum. From the bone marrow and spleen, 2 × 10^6^ cells were aliquoted. All samples were stained with CD11b-eF450 (1:160), Ly6G-PE-Cy7 (1:160), CD115-PE (1:160), CD45-FITC (1:80), DX5-APC-Cy7 (1:20) (all from Invitrogen, Waltham, MA, USA), CD117-APC (1:53), B220-APC-Cy7 (1:160), and CD3e-APC-Cy7 (1:20) (all from BD Biosciences, Franklin Lakes, NJ, USA) for >45 min on ice in the dark. Dead cells were stained with 1 µL of 7-AAD (BD Biosciences) per 200 µL of suspension. The samples were analyzed using a Cytoflex LX, CytExpert software (Beckman Coulter, Brea, CA, USA), and FlowJo (Treestar Inc., San Carlos, CA, USA).

### 4.9. Characterization of Bone Marrow Stem and Progenitor Cells

Lineage depletion (Mouse Hematopoietic Progenitor Cell Enrichment Set, #558451; BD Biosciences, Franklin Lakes, NJ, USA) was performed according to the manufacturer’s protocol. In brief, bone marrow cells were stained with the Biotin Mouse Lineage Depletion Cocktail (CD3e, B220, Gr-1, TER119, and CD11b), followed by magnetic labeling with Streptavidin Particles Plus. Lineage-positive cells were separated using a magnet, with the negative fraction remaining in suspension. The lineage-negative cells were then stained with streptavidin-APC-Cy7 (BD Biosciences) for 15 min, split into two aliquots, and stained for hematopoietic stem cells (CD48-FITC (1:40; BioLegend, San Diego, CA, USA), CD150-BV421 (1:10; BioLegend), Ly-6A/E-PE-Cy7 (1:20; Invitrogen), and CD117-APC (1:160, BD Biosciences)) or hematopoietic progenitor cells (CD16/32-eF450 (1:80; Invitrogen), Ly-6A/E-Pe-Cy7 (1:20; Invitrogen), CD34-PE (1:10; BD Biosciences), and CD117-APC (1:160; BD Biosciences)) for >45 min on ice. Dead cells were stained with 1 µL of 7-AAD (BD Biosciences) per 200 µL of suspension. Samples were analyzed by flow cytometry as described above.

### 4.10. Statistical Analysis

Statistical analyses were performed using the GraphPad Prism 10 software (GraphPad Software Inc., San Diego, CA, USA). Significances were calculated by performing two-tailed Student’s *t*-tests for the following three independent comparisons: (1) WT vs. *Mmp12* KO (^†^ *p* < 0.05, ^††^ *p* ≤ 0.01, and ^†††^ *p* ≤ 0.001), (2) WT vs. *Lal* KO (* *p* < 0.05, ** *p* ≤ 0.01, and *** *p* ≤ 0.001), and (3) *Lal* KO vs. *Lal/Mmp12* DKO (^#^ *p* < 0.05, ^##^ *p* ≤ 0.01, and ^###^ *p* ≤ 0.001). Data are presented as mean values + SD.

## 5. Conclusions

LAL-D is characterized by multiple progressive pathologies, including immune dysfunction. We have shown that while deletion of Mmp12 in Lal KO mice partially reverses certain immune alterations, it does not substantially attenuate the broader pathology associated with LAL-D. In particular, the typical hallmarks of LAL-D, such as hepatomegaly and adipose tissue loss, remained largely unchanged in *Lal/Mmp12* DKO mice, indicating that MMP-12 does not play a central role in these aspects of disease progression. The improvements in thymus morphology and a slight reversal of the lymphoid-to-myeloid shift did not translate into detectable benefits to overall lymphocyte pathology. Accordingly, our data suggest that the contribution of MMP-12 to LAL-D is limited, and modulation of expression/activity is not a feasible therapeutic target. Further studies are required to evaluate potential mechanisms by which MMP-12 affects CD11b+ Ly6G+ cells.

## Data Availability

The data presented in this study are available upon request from the corresponding author. Reagents and detailed methods of all procedures are provided in Section 4 of this manuscript or cited accordingly.

## Supplementary Materials

The supporting information can be downloaded at: https://www.mdpi.com/article/10.3390/ijms252011001/s1.
