# Supplementary material for: Limited Alleviation of Lysosomal Acid Lipase Deficiency by Deletion of Matrix Metalloproteinase 12
Source: Int J Mol Sci. 2024 Oct 13;25(20):11001. doi: 10.3390/ijms252011001 (PMC11506919; doi:10.3390/ijms252011001)
Supplement: Supplementary file 1 [file ijms-25-11001-s001.zip › ijms-3206910-supplementary.pdf]

SUPPLEMENT

Limited Alleviation of Lysosomal Acid Lipase Deficiency by Deletion of Matrix Metalloproteinase 12

Martin Buerger, Melina Amor, Alena Akhmetshina, Valentina Bianco, Bianca Perfler, Armin Zebisch, Thomas Weichhart, Dagmar Kratky

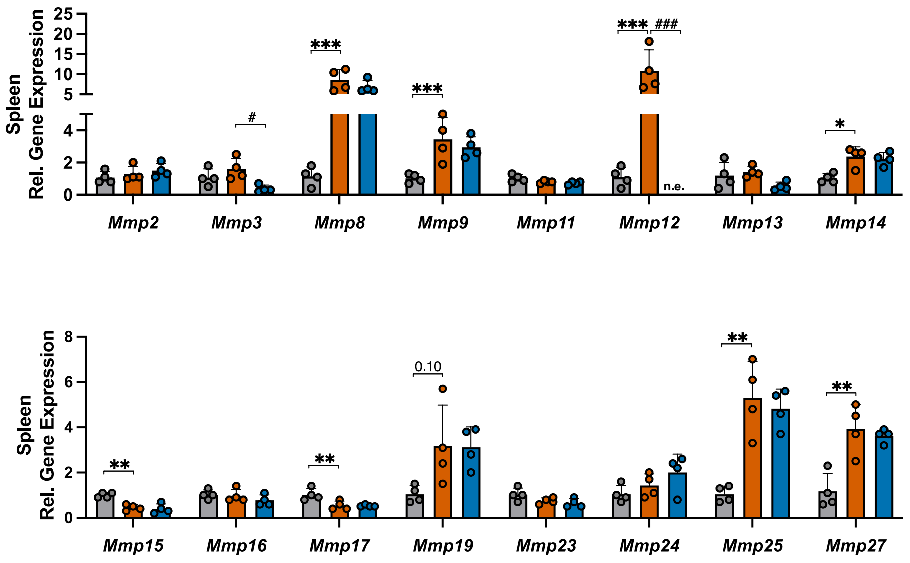

Figure S1: No compensatory upregulation of other MMP family members in the spleen of *Lal/Mmp12* DKO mice. Gene expression of MMP family members in the spleen of 29-30-week-old chow diet-fed male WT, *Lal* KO, and *Lal/Mmp12* DKO mice. Data are shown as means (n = 4) + SD. *p ≤ 0.05, **p ≤ 0.01, and ***p ≤ 0.001 for the comparison between WT and *Lal* KO mice; $^{\#}$p < 0.05 and $^{\#\#\#}$p ≤ 0.001 for the comparison between *Lal* KO and *Lal/Mmp12* DKO mice.

Supplementary Table S1: Complete blood counts of WT, *Mmp12* KO, *Lal* KO, and *Lal/Mmp12* DKO mice.

	WT			*Mmp12* KO			*Lal* KO			*Lal/Mmp12* DKO			t-test (Lal KO vs DKO)
**PLT**	1077	+/-	337	1035	+/-	196	1913	+/-	375	1861	+/-	460	0.84
**MPV**	4.4	+/-	0.58	4.4	+/-	0.16	4.2	+/-	0.16	4.6	+/-	0.30	**0.039**
**PCT**	0.4	+/-	0.11	0.5	+/-	0.09	/	+/-		/	+/-		/
**PDW**	16.3	+/-	0.55	16.2	+/-	0.11	15.9	+/-	0.19	16.1	+/-	0.28	0.15
**RBC**	9.9	+/-	0.68	9.6	+/-	0.78	9.3	+/-	1.06	11.0	+/-	0.94	**0.022**
**MCV**	45.0	+/-	1.66	44.1	+/-	1.02	30.9	+/-	3.05	29.4	+/-	1.64	0.35
**HCT**	0.4	+/-	0.03	0.4	+/-	0.03	0.3	+/-	0.01	0.3	+/-	0.02	**0.0094**
**HGB**	13.9	+/-	1.53	13.8	+/-	0.42	9.0	+/-	0.37	10.4	+/-	1.00	**0.017**
**MCH**	14.0	+/-	1.42	14.5	+/-	1.10	9.6	+/-	0.79	9.4	+/-	0.62	0.62
**MCHC**	32.6	+/-	1.51	32.9	+/-	2.17	31.5	+/-	1.24	32.3	+/-	1.12	0.29
**% RDW**	15.9	+/-	0.82	15.5	+/-	0.31	21.6	+/-	2.25	22.4	+/-	1.42	0.50
**WBC**	12.5	+/-	1.90	9.9	+/-	1.56	14.5	+/-	1.83	16.8	+/-	5.52	0.37
**LYMF**	9.7	+/-	1.62	7.6	+/-	1.25	5.4	+/-	1.37	7.1	+/-	2.63	0.21
**GRAN**	2.5	+/-	0.55	2.0	+/-	0.59	8.4	+/-	0.88	8.7	+/-	2.99	0.84
**MON**	0.3	+/-	0.07	0.3	+/-	0.08	0.6	+/-	0.21	1.0	+/-	0.39	0.11
**% LYN**	0.77	+/-	0.04	0.77	+/-	0.04	0.37	+/-	0.06	0.42	+/-	0.05	0.19
**% GRA**	0.20	+/-	0.04	0.20	+/-	0.04	0.59	+/-	0.06	0.53	+/-	0.06	0.11
**% MON**	0.03	+/-	0.01	0.03	+/-	0.01	0.05	+/-	0.01	0.06	+/-	0.01	0.085
**% EOS**	0.0	+/-	0	0.0	+/-	0	0.0	+/-	0	0.0	+/-	0	/

Supplementary Table S2: List of primers used for real-time PCR.

Gene	Forward Sequence 5´-3´	Reverse Sequence 5`-3`
B220	CCAGTGATGGTGTGTTATCCAC	GGGGGTATCAACAGGAAAGGC
Ccl2	TTAAAAACCTGGATCGGAACCAA	GCATTAGCTTCAGATTTACGGGT
Cd3	GGTGCTCCAGGATTTCTCGG	GCCTTGGCCTTCCTATTCTTG
Cd68	AACAGGACCTACATCAGAGC	TCAAGGTGAACAGCTGGAGA
Cxcl1	CTGGGATTCACCTCAAGAACATC	CAGGGTCAAGGCAAGCCTC
Cxcl2	AGTGAACTGCGCTGTCAATG	GCCCTTGAGAGTGGCTATGA
Cxcl5	AGCGGTTCCATCTCGCCATTC	CTCCGTTGCGGCTATGACTG
Cxcr1	TCTGGACTAATCCTGAGGGTG	GCCTGTTGGTTATTGGAACTCTC
Cxcr2	ATGCCCTCTATTCTGCCAGAT	GTGCTCCGGTTGTATAAGATGAC
Cxcr4	GACTGGCATAGTCGGCAATG	AGAAGGGGAGTGTGATGACAAA
Elane	CAGGAACTTCGTCATGTCAGC	AGCAGTTGTGATGGGTCAAAG
Emr1	CTTTGGCTATGGGCTTCCAGTC	GCAAGGAGGACAGAGTTTATCGTG
Hprt	GTTGGGCTTACCTCACTGCT	TAATCACGACGCTGGGACTG
Il1b	GAAATGCCACCTTTTGACAGTG	TGGATGCTCTCATCAGGACAG
Ly6g	TGCCCCTTCTCTGATGGATT	TGCTCTTGACTTTGCTTCTGTGA
Mmp12	CTGCTCCCATGAATGACAGTG	AGTTGCTTCTAGCCCAAAGAAC
Ppia	GAGCTGTTTGCAGACAAAGTTC	CCCTGGCACATGAATCCTGG
Tnf	CCACCACGCTCTTCTGTCTAC	AGGGTCTGGGCCATAGAACT
